# Dissociating visuo-spatial and verbal working memory: It’s all in the features

**DOI:** 10.3758/s13421-018-0882-9

**Published:** 2018-12-17

**Authors:** Marie Poirier, James M. Yearsley, Jean Saint-Aubin, Claudette Fortin, Geneviève Gallant, Dominic Guitard

**Affiliations:** 1 Department of Psychology, City, University of London, Northampton Square, London, EC1V 0HB UK; 2 Université de Moncton, Moncton, NB Canada; 3 Laval University, QC, Québec Canada

**Keywords:** Working memory, Memory models, Short term memory

## Abstract

Echoing many of the themes of the seminal work of Atkinson and Shiffrin (*The Psychology of Learning and Motivation,* 2; 89–195, 1968), this paper uses the feature model (Nairne, *Memory & Cognition, 16,* 343–352, 1988; Nairne, *Memory & Cognition, 18;* 251–269, 1990; Neath & Nairne, *Psychonomic Bulletin & Review*, *2*; 429–441, 1995) to account for performance in working-memory tasks. The Brooks verbal and visuo-spatial matrix tasks were performed alone, with articulatory suppression, or with a spatial suppression task; the results produced the expected dissociation. We used approximate Bayesian computation techniques to fit the feature model to the data and showed that the similarity-based interference process implemented in the model accounted for the data patterns well. We then fit the model to data from Guérard and Tremblay (2008, *Journal of Experimental Psychology: Learning, Memory, and Cognition, 34*, 556–569); the latter study produced a double dissociation while calling upon more typical order reconstruction tasks. Again, the model performed well. The findings show that a double dissociation can be modelled without appealing to separate systems for verbal and visuo-spatial processing. The latter findings are significant as the feature model had not been used to model this type of dissociation before; importantly, this is also the first time the model is quantitatively fit to data. For the demonstration provided here, modularity was unnecessary if two assumptions were made: (1) the main difference between spatial and verbal working-memory tasks is the features that are encoded; (2) secondary tasks selectively interfere with primary tasks to the extent that both tasks involve similar features. It is argued that a feature-based view is more parsimonious (see Morey, 2018, *Psychological Bulletin*, *144*, 849–883) and offers flexibility in accounting for multiple benchmark effects in the field.

This paper presents new data examining interference effects in working-memory tasks for visuo-spatial and verbal material. Interference effects were produced by using classic dual-task techniques where participants encode one stream of stimuli while simultaneously completing an interference task (e.g. continuously mouthing the words “tea time, tea time, tea time” or tapping squares in a repeated pattern).

We argue that the results in this article can be handled with a feature-based model. There are many feature-based models, including those that grew from the Atkinson and Shiffrin (1968) chapter, such as SAM (search of associative memory; Raaijmakers & Shiffrin, 1980) and REM (retrieving effectively from memory; Shiffrin & Steyvers, 1997). We decided to use a feature model (FM hereafter; Nairne, 1988, 1990; Neath, 1999; Neath & Nairne, 1995) which is from the short-term memory literature and with which we were more familiar. The FM successfully accounts for numerous benchmark effects in the *verbal* working-memory literature through simulations that reproduce the relevant patterns. Importantly, this paper is the first to quantitatively fit the FM to data. The model is fit to the data of Experiment 1, and then the generality of the approach is tested by using a similar set of assumptions and parameters to model a more complex set of findings (previously published by Guérard & Tremblay, 2008). For the first time, also, the FM is used to account for performance in visuo-spatial and verbal tasks, in both single-task and dual-task conditions. Central to the aims of the paper, this is accomplished without calling upon separate visuo-spatial and verbal modules (e.g. the working-memory model; Baddeley, 1986, 2012; Baddeley & Hitch, 1974). The absence of domain-specific modules in our approach is worth highlighting, as the dominant view in the working-memory field proposes separate visuo-spatial and verbal processing structures (see C. C. Morey, 2018, for a thorough discussion of this point).

The ideas relied on here—as well as the general strategy adopted—echo many of the themes and issues tackled by the seminal paper by Atkinson and Shiffrin (1968). Let us mention a few. As is well known, Atkinson and Shiffrin (1968) proposed a memory model with three structural components: a sensory register, a short-term store, and a long-term store. Major sections in their paper focused on the interplay between the short-term and long-term stores. The FM also proposes separate short-term and long-term stores, with the short-term store holding the cues used to probe the long-term store. In addition, as in Atkinson and Shiffrin, there is a distinction between the memory structures involved and the nature of the experimental tasks: working-memory performance is assumed to involve both stores, even if the experiments rely on short-term or working-memory *tasks*. Further, the FM calls upon more than one ‘copy’ or ‘trace’ of the to-be-remembered information; it is assumed that studying an item sets up a representation both in short-term and long-term memory, echoing similar proposals in Atkinson and Shiffrin. The FM assumes that the presentation of an item creates a multicomponent ‘trace’; representations are thought of as vectors of features that can vary in type and number; some of these represent the physical features of the presentation while others are related to the features generated by, for example, the recognition or categorization of the item. In the words of Atkinson and Shiffrin, “Our preference is to consider the trace as a multicomponent array of information (p. 102).” As explained later, this is a central dimension of the FM; it allows us to model the experimental patterns examined here. Finally, Atkinson and Shiffrin emphasized the importance of control processes, such as rehearsal buffers (we return to this suggestion in the General Discussion, when reviewing the findings and modelling results).

There are some differences in assumptions and approach, of course; these reflect the current preoccupations in working-memory research, the knowledge that has accumulated in the past decades, as well as other issues. For instance, although decay from the short-term store was an important mechanism in the work of Atkinson and Shiffrin (1968), in the FM, information loss in the short-term store happens through retroactive interference (as is other recent models in the field, e.g. Farrell et al., 2016; Oberauer & Lin, 2016). Interference is similarity-based in the FM while decay was attributed to capacity limits in Atkinson and Shiffrin. Finally, the emphasis in our work was to explain performance across verbal and visuo-spatial tasks, whereas in Atkinson and Shiffrin (1968) the focus was more on what they called the auditory-verbal-linguistic short-term store.

## Dissociations, coding, and working memory

Dissociations occupy a significant role in the development of psychological models and theories of cognition (Baddeley, 2003; Dunn & Kirsner, 1988, 2003; Henson, 2006; C. C. Morey, 2018). Two general dissociation types are more typically discussed. A *single dissociation* occurs when Factor A selectively affects performance on a given task (e.g. it affects Task 1 but has no effect on Task 2); a single dissociation also occurs when Factor A has an effect on a task while Factor B does not. Moreover, a *double dissociation* occurs when Factor A affects performance on Task 1, but not on Task 2, while Factor B produces the reverse pattern.

Double dissociations or multiple single dissociations are frequently interpreted as indicating that multiple systems or structures are supporting performance. For instance, dissociations have been called upon to argue in favour of a distinction between short-term and long-term memory, episodic and semantic memory, procedural and declarative memory, and implicit and explicit memory (e.g. Norris, 2017; Schacter, 1987; Squire, 1994). Moreover, dissociations in patterns of brain activation are typically taken to imply that separate systems are involved in the tasks producing said patterns (D’Esposito, 2007). However, these interpretations have been challenged by other views, where, for example, a single system produces the dissociations (Hintzman, 1984. 1986; Murdock, 1993), or where a transfer-appropriate processing analysis can account for the findings (Crowder, 1993; Roediger, Weldon, & Challis, 1989). This is also true of differing activation patterns uncovered, for example, within studies relying on fMRI (Henson, 2006; Lewandowsky, Ecker, Farrell, & Brown, 2012; for a robust critique of modularization in neuroscience, see Uttal, 2011). Hence, when interpreting dissociations, alternative hypotheses to modularity are a possibility, but they are often overlooked.

In the working-memory field, the dominant model is the working-memory model (Baddeley, 2012; Baddeley & Hitch, 1974). The model proposes separate short-term structures for visuo-spatial and verbal information (i.e. domain-specific mechanisms). In her recent review, C. C. Morey (2018) described the model as “one of the most influential theoretical frameworks in cognitive psychology” (p. 849); nevertheless, she is very critical of said modular approach. C. C. Morey (2018) reviews the data on both the experimental and neuropsychological dissociations that support the proposal and concludes that the idea of distinct domain-based modules should be reconsidered; she suggests that future theorizing should focus on testing more parsimonious accounts that explain domain-specific interference without calling upon distinct modules.

The work reported herein investigates one such account. More specifically, we asked if the dissociations typically taken to support different verbal and spatial working memory systems can be accounted for by assuming coding differences between tasks (Guérard & Tremblay, 2008). To this aim, we called upon the FM, a computational model developed to account for working memory phenomena.

With respect to short-term/working-memory models, Crowder (1993) argued that one of the problems is that structure and coding are often confounded. Applying Crowder’s logic to the working-memory approach of Baddeley and his collaborators, it can be argued that there is a confound between verbal and visuo-spatial *coding* on the one hand, and verbal and visuo-spatial memory modules on the other. If one assumes this redundancy is not necessary, then it should be possible to demonstrate that differences in coding are sufficient to produce typical dissociations.

Guérard and Tremblay (2008) developed a similar argument. After reviewing the data relevant to the debate about the modularity of working memory, they showed that a detailed analysis of error patterns in immediate memory performance supports more unitary views. Moreover, in their second experiment they provided an example of a double dissociation. In this study, a spatial secondary task interfered with a spatial primary task, while having a reduced effect on a verbal task. Conversely, a verbal secondary task showed the reverse pattern, as it had a negative impact on the verbal primary task, while having no effect on the spatial primary task. The authors argued that the complete set of data could be explained by a unitary view if a similarity-based interference assumption is adopted. The implication was that spatial primary and secondary tasks generate similar codes or features and hence interfere with each other; a similar argument can be applied to the verbal tasks. As the features involved in the spatial and verbal tasks are less similar, they would not interfere with each other as much.

In the present paper, we completed a systematic examination of these ideas by fitting the FM to data from our own dual-task experiment as well as to the data provided by Guérard and Tremblay (2008). The FM was selected as the similarity-based interference on which the model relies seemed to suggest it would be well-fitted to our aims. Furthermore, the FM is a prominent model in the working memory literature. The familiarity of the authors with the model also played a role in the choice. Other working-memory models that incorporate feature-based representations and similarity-based interference (e.g. C-SOB; Lewandowsky & Farrell, 2008) could perhaps also account for the findings. Interestingly, models that derived from the original Atkinson and Shiffrin (1968) paper, such as SAM (search of associative memory; Raaijmakers & Shiffrin, 1980) could also no doubt handle general dissociations of the type described here, provided a means of encoding and retrieving serial order was added to the proposal. The idea that more than one feature-based model could handle verbal/visuo-spatial dissociations strengthens the general argument made here: It is possible to account for complex dissociation patterns without proposing separate systems for visual and verbal short-term memory.

## The feature model

In the FM, items are represented by two types of features. On one hand, encoding is thought to generate modality-dependent features, related to physical presentation conditions such as font size or voice quality. On the other hand, items also produce modality-independent features, generated by internal processes of categorization and identification. Furthermore, within the FM, items simultaneously generate traces in short-term and long-term memory (called primary and secondary memory). In both cases, items are represented by vectors of features, with each (randomly generated) feature typically taking on a value of +1 or −1.

Traces in primary memory are subject to degradation through overwriting by the following item. This retroactive interference process is similarity-based: If Feature 5 of Item « *n* » is identical to Feature 5 of Item « *n* − 1 », then this feature of Item « *n* − 1 » will be overwritten (set to 0). In contrast, representations in long-term memory are assumed to remain intact.

At the point of recall, the correspondence between each degraded primary memory trace and the set of relevant traces in secondary memory is computed. The secondary memory trace with the highest *relative* similarity to the primary memory trace being considered has the highest probability of being recalled. The selection of the secondary memory trace to recall is based on a similarity-based choice rule (cf. Luce, 1963), as follows:

$$ Ps\ \left( SMj\ |\  PMi\right)=\frac{s\left(i,j\right)}{\sum s\left(i,k\right)}, $$

where the conditional probability that the secondary memory trace *SM*_*j*_ will be sampled, given the primary memory trace *PM*_*i*_, depends on a similarity ratio; *s*(*i, j*) is the computed similarity between *PM*_*i*_ and *SMj,* and *Σs*(*i, k*) is the summed similarity between *PM*_*i*_ and all relevant secondary memory traces.

Similarity is related to the feature-to-feature correspondence between primary and secondary traces. This relationship calls upon a calculation of the psychological distance between the two traces, based on a function described by Shepard (1987):

$$ s\left(i,j\right)={e}^{-{d}_{ij}} $$

This distance, *d*_*ij*_, is simply calculated by adding the number of mismatched features, *M*, and dividing by the number of compared features, *N*, as in:

$$ {d}_{ij}=\frac{a}{N}\sum {b}_k{M}_k, $$

where *a* is a scaling constant, and *b*_*k*_ is an attention bias parameter that is set to 1 for all simulations here (and hence has no influence on results). Output interference is included in the model, by assuming recovery from the *SM* set is related to prior recall of the item, in the following manner:

$$ {P}_r={e}^{- cr}, $$

where « *c* » is a scaling constant and « *r* » is the number of times a sampled item has already been recalled.

The FM also has a mechanism to account for order memory (Neath, 1999). To achieve this, aspects of Estes’s (1972, 1997) perturbation model were incorporated into the model. The suggestion was that item positions were encoded in primary memory and that positional information drifts (or perturbs) along the positional dimension as time intervals pass. The probability that an item will move along the positional dimension is given by ***θ***, which is typically set to .05 and not considered a free parameter. For simplicity, it is assumed that perturbations are equally likely in either direction (but see Poirier, Saint-Aubin, Mair, Tehan, & Tolan, 2015). It is further assumed that an item cannot drift beyond the start and end positions (e.g. an item cannot drift beyond the first or last position). Specifically, the probability that an item, *I*, will move to a position, *p*, during the next interval, t + 1, is given by

$$ {I}_{p,t+1}=\left(1-\theta \right){I}_{p,t}+\left(\frac{\theta }{2}\right){I}_{p,1+t}+\left(\frac{\theta }{2}\right){I}_{p,1+t} $$

For boundary items, slightly modified equations are used. For Position 1, the first equation below is applied whereas for the last position, *n*, the second equation is employed:

$$ {\displaystyle \begin{array}{c}{I}_{1,t+1}=\left(1-\frac{\theta }{2}\right){I}_{1,t}+\left(\frac{\theta }{2}\right){I}_{2+t}\\ {}{I}_{n,t+1}=\left(1-\frac{\theta }{2}\right){I}_{n,t}+\left(\frac{\theta }{2}\right){I}_{n-1,t}\end{array}} $$

Incorporating order adds a free parameter to the FM; this is ***π***, the number of opportunities to perturb which, for each item, is equivalent (for simplicity). With the above, the FM generates positional uncertainty distributions; these describe the probability that an item was initially encoded as being in each serial position (i.e. what are the chances that the first item presented was encoded as being in Position 1, 2, 3, . . . *n*). At the point of retrieval, the item that has the highest probability of being in the first position will be used first; then, the item with the greatest probability of being in the second position will be used second, and so on. As movement in either direction is possible (and random), most will not drift far from their original position, as is typically observed in the data (e.g. Nairne, 1991). Hence, to summarize, this view of order encoding implies that order errors occur due to a gradual loss of precision in positional coding. The loss of precision described by the perturbation equations produces the same pattern of position error gradients as observed in empirical data (e.g. Estes, 1972, 1997; Nairne, 1991); order information is encoded and preserved in primary memory, but order cues lose precision.

## Accounting for working-memory findings

As mentioned previously, the FM model accounts for many of the benchmark effects in the short-term memory literature. To illustrate how the model operates, a few of these effects, as well as the way the FM handles them, are considered below.

The inception of the FM was motivated by an attempt to account for the modality effect, observed in immediate serial recall (Corballis, 1966). The modality effect refers to the fact that recall is superior when items are presented aurally as opposed to visually. In fact, the difference is attributable to a larger recency effect for auditory items (Conrad & Hull, 1968; Murray, 1966; Nairne, 1988). To account for this effect, the FM assumes that auditory traces have a greater number of modality dependent features than traces generated through visual presentation. Nairne (1990) noted that this assumption is consistent with the literature indicating that interference based on visual characteristics is not typically found within immediate serial recall performance. He also noted that the evidence for speech-like coding in primary memory with visual presentation is extensive and suggests that, without auditory cues, we tend to rely on modality independent (inner voice) features to represent items. Hence, in the FM, it is the greater number of modality-dependent features, associated with auditory presentation, that leads to an auditory advantage for the recency position. This occurs because the model posits that the last item of a list is followed by internally generated activity, which overwrites modality-independent features while leaving modality-dependent features intact. The recall of the last item from an aurally presented list will benefit from these intact modality-dependent features. The reason for this is that the intact features serve to increase the correspondence between the degraded primary memory trace of the last item and its secondary memory counterpart. Conversely, because visually based traces do not have many modality dependent features, there is a smaller recency effect for these items.

A related effect is the suffix effect (Dallett, 1965). The latter refers to the detrimental effect of an auditory item that follows an aurally presented list. If, for example, the word « recall » is heard after the last item, the recency advantage or modality effect is much reduced. The same auditory suffix does not affect the recall of visually presented items. The FM easily accounts for this effect by assuming the suffix overwrites the modality dependent features of the last item, obviating the advantage attributed to these features. Because the recall of visually presented items relies on modality independent features—few modality-dependent features being encoded—the suffix will not have a significant effect on performance in this case.

Another benchmark effect in the working memory literature is the phonemic similarity effect; there is a decrement in performance observed when to-be-recalled items share phonemic features (Conrad, 1964). Again, because the FM assumes a similarity-based interference mechanism, it handles this effect easily. Phonemically similar lists are associated with a decrease in performance because relative to phonemically distinct lists, there will be more overwriting for these items.

Finally, as a last example of the FM’s operation, consider the effect of articulatory suppression. When a participant engages in articulatory suppression, he or she simply repeats an irrelevant word or syllables (e.g. “a-b-c-d-a-b-c-d”) during list presentation. Articulatory suppression reduces immediate serial recall performance considerably, and it interacts with the previously discussed effects in complex ways. The FM accounts for the influence of this dual task, as well as its interactions with the modality, suffix, and phonemic similarity effects. This is achieved through the feature adoption assumption (Neath, 1999). In feature adoption, the repeated production of irrelevant sounds produces similar features that are incorporated into each item’s traces. The net effect of this process is to increase the similarity of the items in the suppressed condition, which reduces performance.

## Experiment 1

Experiment 1 investigates the effects of spatial suppression and articulatory suppression on the visuo-spatial and verbal-control versions of the Brooks matrix task (Brooks, 1967). Spatial suppression is a visuo-spatial analog to articulatory suppression, and there is evidence showing it selectively influences performance of visuo-spatial tasks (Borst, Niven, & Logie 2012; Farmer, Berman, & Fletcher, 1986; Logie, 1995). The predicted pattern was a single dissociation. We expected that spatial suppression would disrupt the visuo-spatial task, while having little or no effect the verbal version. With respect to articulatory suppression, because the material for both tasks was presented verbally, we expected an effect in both cases. We included a 10-second delay after the presentation of the to-be-recalled items; the rationale was that this would increase the potential impact of the secondary tasks in the relevant conditions. Pilot testing was used to adjust list length and performance levels.

### Method

#### Participants

Forty-eight participants (34 women, 14 men) completed the experiment; their mean age was 21.4 years (range: 17–46). Half of the participants were assigned to the visuo-spatial version of the Brooks matrix task and the other half to the verbal version. They were volunteers (psychology undergraduates), and none had participated in this type of experiment before.

#### Tasks and materials–Primary tasks

Stimuli presentation was controlled by a computer running a bespoke program developed with E-Prime (Psychology Software Tools, Inc., Version 2). In the visuo-spatial version of the Brooks matrix task, participants were to visualize a 4 × 4 matrix. To support this visualization, a 4 × 4 matrix was presented on screen for the first 3 s of each trial; it was replaced by a white screen and the auditory instructions then started. There were practice trials described in the procedure below. The second square of the second row was designated as the starting square. On each trial, participants were to imagine a path within the 4 × 4 grid based on eight aurally presented sentences, as in the following example: “In the starting square, put a 1; in the next square to the right put a 2; in the next square down put a 3; in the next square to the right put a 4; in the next square up put a 5; in the next square left put a 6; in the next square to the up put a 7, in the next square left put an 8”. As the first sentence is always the same, the number of items to remember was seven. Each sequence or path contained one cross-over or contact point, that is, if one drew a line following the described path, one of the squares would be used twice; this was done to make the series of approximately comparable difficulty. At the point of recall, participants were to say aloud, in strict serial order, the words describing the correct path around the matrix (right, left, up, down; because participants were French speaking, the words used were *droite, gauche, haut, bas*). In the control verbal version of the task, each trial comprised six sentences (five items to recall) with precisely the same structure as above, except that the words “right, left, up, and down” were replaced by the nonspatial adjectives “quick, slow, good, and false” (actual words: *vite, lent, bon, faux*). The verbal version of the Brooks task is the most difficult one (Brooks, 1967). Hence, the number of sentences for this material was reduced to equate performance levels on both tasks and avoid floor/ceiling effects. At recall, the target words were again said aloud, in their order of presentation. Responses were digitally recorded and later scored. The sentences were developed using the text-to-speech website Oddcast (http://www.oddcast.com/technologies/tts/) using Leo’s voice. They were adapted using the SoundTap (NCH Software, Version 3.04) and Audacity (Audacity, 2.1.0) packages so that each sentence was presented within a 3-second envelope; this was done by adding silence to any sentence that lasted less than 3 seconds (no sentence lasted more than 3 seconds). Hence, sentence presentation proceeded at a rhythm of one sentence every 3 seconds.

#### Secondary tasks

**Fig. 1:**
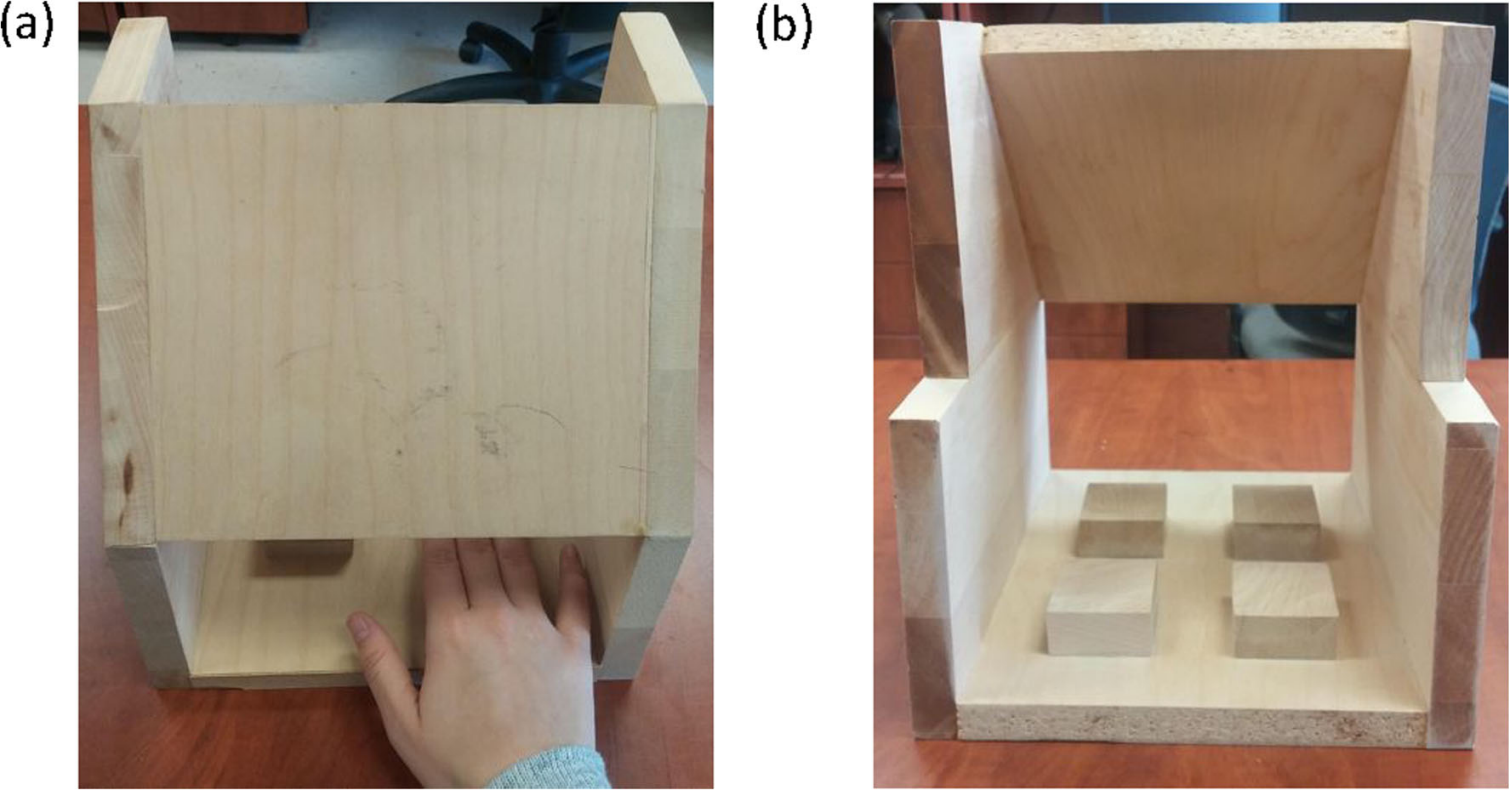
Apparatus used for the spatial tapping task (**a**) seen from the participant’s perspective and (**b**) seen from the back and the perspective of the camera

For the spatial suppression task (Farmer et al., 1986), participants used their dominant hand to sequentially tap four 5 cm × 5 cm wooden blocks, placed in a square arrangement, and hidden from view (see Fig. 1a–b); tapping was counterclockwise at a rhythm of approximately 2 taps per second. A camera at the back of the apparatus filmed the tapping and participants were aware of this. In the articulatory suppression condition, participants repeated the word *mathématiques* continuously, producing about three utterances every 2 seconds. In the secondary task conditions, spatial and articulatory suppression continued throughout the trial, which included a 10-second interval following the presentation of the sentences.

#### Design and procedure

The forty-eight participants were randomly assigned to one of two experimental groups. Group 1 was tested with the verbal material while Group 2 was tested with the visuo-spatial matrix task.

Participants were individually tested in a session of approximately 1 hour. Each session started with familiarization trials. For those assigned to the visuo-spatial matrix task, there was a two-part familiarization procedure. The first part involved five typical trials. However, instead of attempting to recall the target direction words, they were asked to draw the path that the sentences described within a matrix drawn on a sheet of paper; the starting square was marked (second square of the second row). This was done to encourage participants to use a visuo-spatial strategy to encode the aurally presented instructions during the experimental trials. For the second part of the familiarization procedure, four standard single-task trials were presented. For those participants taking part in the verbal version of the task, the familiarization procedure involved completing four standard trials. Participants then completed three blocks of 14 trials; one block involved the primary task on its own, one block comprised the primary task with spatial suppression and a further block was completed with articulatory suppression. The first trial of each block was a further practice trial. The order of the blocks was counterbalanced using a Latin square.

Each trial began with a 3-second visual warning signal (“Attention”). For the visuo-spatial group, this appeared at the top centre of the screen and was accompanied by a 4 × 4 matrix in the centre of the screen; the matrix was 19.5 cm high × 17 cm wide. For the verbal group, the warning signal appeared in its own in the centre of the screen. In the dual-task conditions, participants began the suppression tasks as soon as the visual warning signal appeared. After 3 seconds, a blank screen appeared, and the primary task sentences were heard through headphones. A 10-second interval then followed; in dual-task trials, participants continued the suppression task throughout this interval. The end of the interval was marked by the appearance of 3 question marks “???” in the centre of the screen; participants then verbally recalled the target words. If they forgot one of the words, they were to say “pass” (in French *passe*). Participants could not backtrack to change a response. Once recall was over, pressing the space bar started the next trial.

### Results

**Fig. 2:**
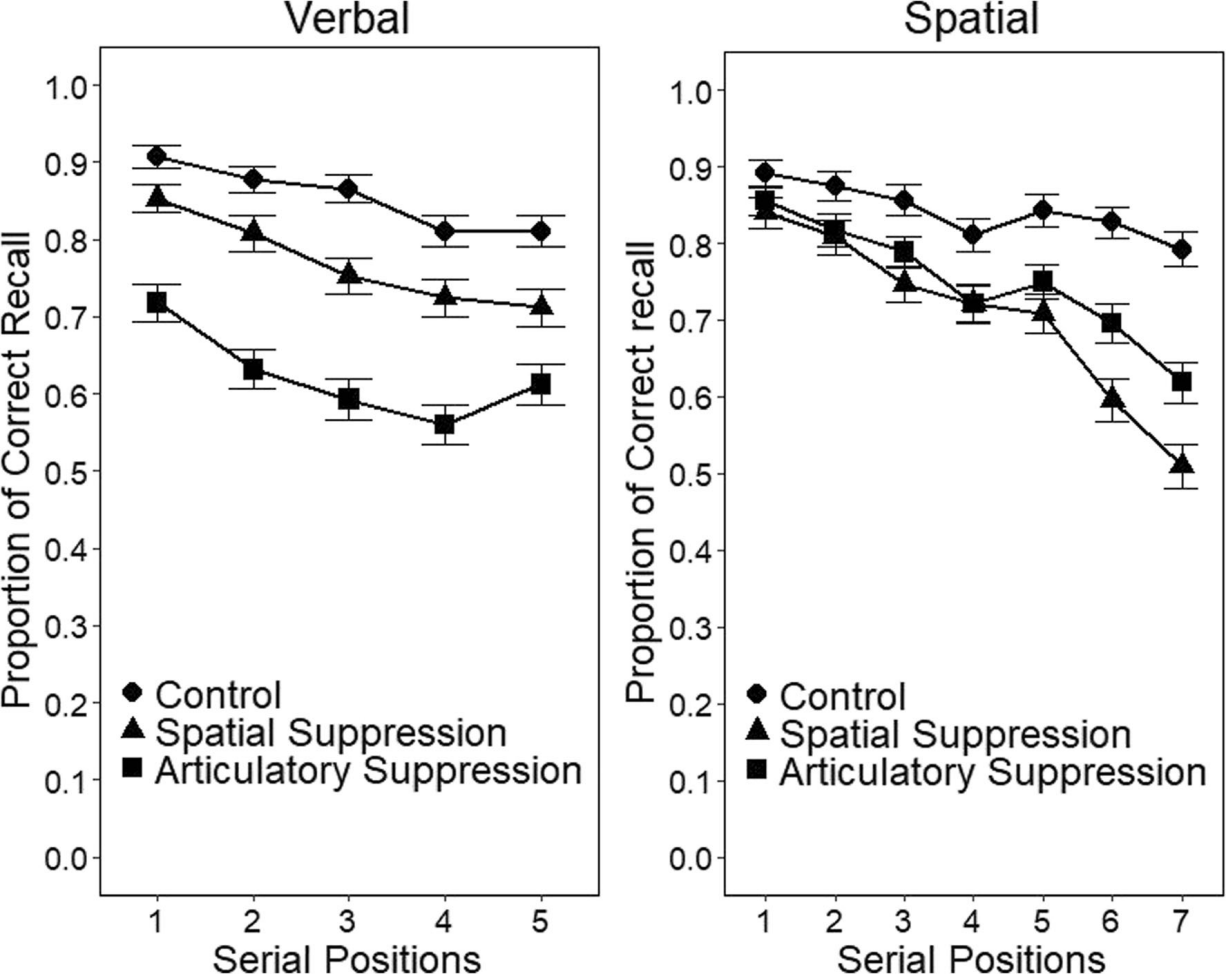
Proportion of correct recall for the Brooks verbal (left panel) and spatial (right panel) matrices as a function of condition (control, spatial suppression, and articulatory suppression) and serial position. Error bars represent 95% within-participant confidence intervals computed according to R. D. Morey’s (2008) procedure

The main performance measure was the number of target words correctly recalled in their studied serial position. Figure 2 presents the means for each condition of the visuo-spatial and the verbal tasks. As expected, both articulatory and spatial suppression had a detrimental effect on the visuo-spatial version of the Brooks matrix task, while in the case of the verbal version, there appears to be a smaller effect of spatial suppression and a more detrimental impact of articulatory suppression.

The data from each task was analysed separately, as serial position was included as a factor and each task had a different number of items per list. For the visuo-spatial task, a 3 (condition: single task, articulatory suppression and spatial suppression) × 7 (serial position) repeated-measures ANOVA produced a significant main effect of condition, *F*(2, 46) = 12.54, η^2^_p_ = .35, *p* < .001, serial position, *F*(6, 138) = 31.25, η^2^_p_ = .58, *p* < .001, and a significant interaction, *F*(12, 276) = 5.27, η^2^_p_ = .19, *p* < .001. Planned contrasts revealed that recall was significantly lower under spatial suppression than in the control condition at all serial positions but the first. However, the detrimental effect of articulatory suppression was limited to the last three serial positions, and spatial suppression was more detrimental than articulatory suppression on the last two serial positions.

For the verbal version of the task, a 3 (condition) × 5 (serial position) repeated-measures ANOVA revealed a main effect of condition, *F*(2, 46) = 24.04, η^2^_p_ = .51, *p* < .001, and of serial position, *F*(4, 92) = 12.80, η^2^_p_ = .36, p < .001. The two-way interaction did not reach significance, *F* < 1. Tukey (HSD) post hoc comparisons indicated that both spatial and articulatory suppression significantly depressed performance; articulatory suppression had a more detrimental effect than spatial suppression on recall.

### Discussion

In this experiment, we expected that both spatial and articulatory suppression would hinder performance of the Brooks visuo-spatial matrix task; spatial suppression was predicted to have an effect because it has spatial requirements, which are thought to also be involved in the primary task. Articulatory suppression was expected to also have an impact as the to-be-remembered material was verbally presented to participants. With respect to the verbal version of the Brooks matrices, a selective effect of articulatory suppression was expected with little or no effect of spatial suppression; the results did show an effect of spatial suppression albeit a significantly weaker one. Although the pattern is not characteristic of a perfect single dissociation, it generally conforms to the main predictions put forward at the outset: both secondary tasks affect the visuo-spatial primary task in a similar fashion while articulatory suppression has more impact than spatial suppression on the verbal version of the primary task. The question we asked was if this reasonably complex set of results could be modelled by assuming that secondary tasks have different levels of similarity (in features) with the two primary tasks.

### Modelling results

Our modelling aims were twofold; firstly, we wished to see whether the FM can provide a good account of the data. In previous work, the model was assessed by looking for qualitative similarity between data and simulations (e.g. Neath, 2000). While this approach can provide some evidence for a particular model, it falls short of the state of the art in model evaluation (Farrell & Lewandowsky 2018). Here, we assessed model performance by directly fitting the model as outlined below. As far as we are aware, this is the first attempt to quantitatively fit the FM to data.

Secondly, we sought to use the model to provide evidence for dissociation in the data. To do this, we compared a version of the FM which allowed for dissociations with one that did not. By computing a Bayes factor for the ‘full’ version of the model over the restricted one, we can provide an alternative measure of the evidence for a dissociation.

### General modelling procedures

Model fitting for both experiments was done using approximate Bayesian computation (ABC; see Marin, Pudlom, Robert, & Ryder, 2012; Turner & Van Zandt, 2012, for a review), using a version of sequential Monte Carlo sampling known as partial rejection control (Sisson, Fan, & Tanaka, 2007), hereafter referred to as ABC-PRC. Full details are given in the Appendix. Our approach was to fit the group data (as opposed to individual participants); however, all data from a given experiment was fit at once, which is more demanding for the model than fitting each condition, since some parameters may be forced to take the same values across conditions.

To accommodate these results with the FM, we maintained as many of the typical settings as possible (e.g. Neath & Nairne, 1995); the aim was to use a version of the model that continues to account for multiple experimental phenomena while also accounting for the current pattern of results. Both articulatory suppression and spatial suppression were taken to affect the primary memory traces through feature adoption. The relevant parameter in the model was the number of similar features, which is an estimate of how many modality independent features are set to +1 as a result of the suppression task, relative to the control condition. This was allowed to vary in the model fits, although we imposed certain constraints on the number of similar features in each condition, which are described in more detail below. Priors for the numbers of similar features were taken to be 20 * Beta (2, 2), reflecting a weak expectation that feature adoption will tend to affect some but not all features.

Feature adoption refers to the idea that information from the dual task will tend to overwrite information from the main (recall) task. It seems reasonable that this process will be more effective when the dual task and the main task are similar. We implemented this by including a parameter that increases the number of similar features in cases where the dual and main tasks are similar, that is, when the main task was spatial recall and the dual task was tapping. This parameter essentially controls the degree of dissociation—if it is zero, then dual tasks are equally disruptive regardless of the nature of the main task, while large values indicate a much greater degree of disruption when the modality matches that of the main task. The prior for this parameter was taken to be uniform on the interval [0, 1], representing no prior assumption about the presence of dissociation.

The other parameters that could vary were the distance scaling functions, which reflect the overall task difficulty. These were allowed to vary with the type of stimuli (verbal, visuo-spatial) but did not vary between conditions—so they can be thought of as reflecting any difficulty difference between primary tasks. Priors for the distance scaling functions were taken to be normal distributions with a mean of 20 and a standard deviation of 10.

As well as the main version of the FM, we also fit a ‘null’ version where the parameter controlling the degree of dissociation was fixed to be zero. This allows us to assess the strength of evidence for dissociations by performing a model comparison between the regular and null models.

To summarize, we assume different suppression tasks impact the memory vectors of the relevant/similar primary task in the same basic way. The fitting process determines which values produced the best fits across all conditions and positions. Each type of suppression task is assumed to have a different impact on the memory vectors, and this is generally reduced when the primary and dual tasks are dissimilar. The data and model code used in our analysis are available on the Open Science Framework project page (https://osf.io/6sae4/).

### Fitting for Experiment 1

**Fig. 3:**
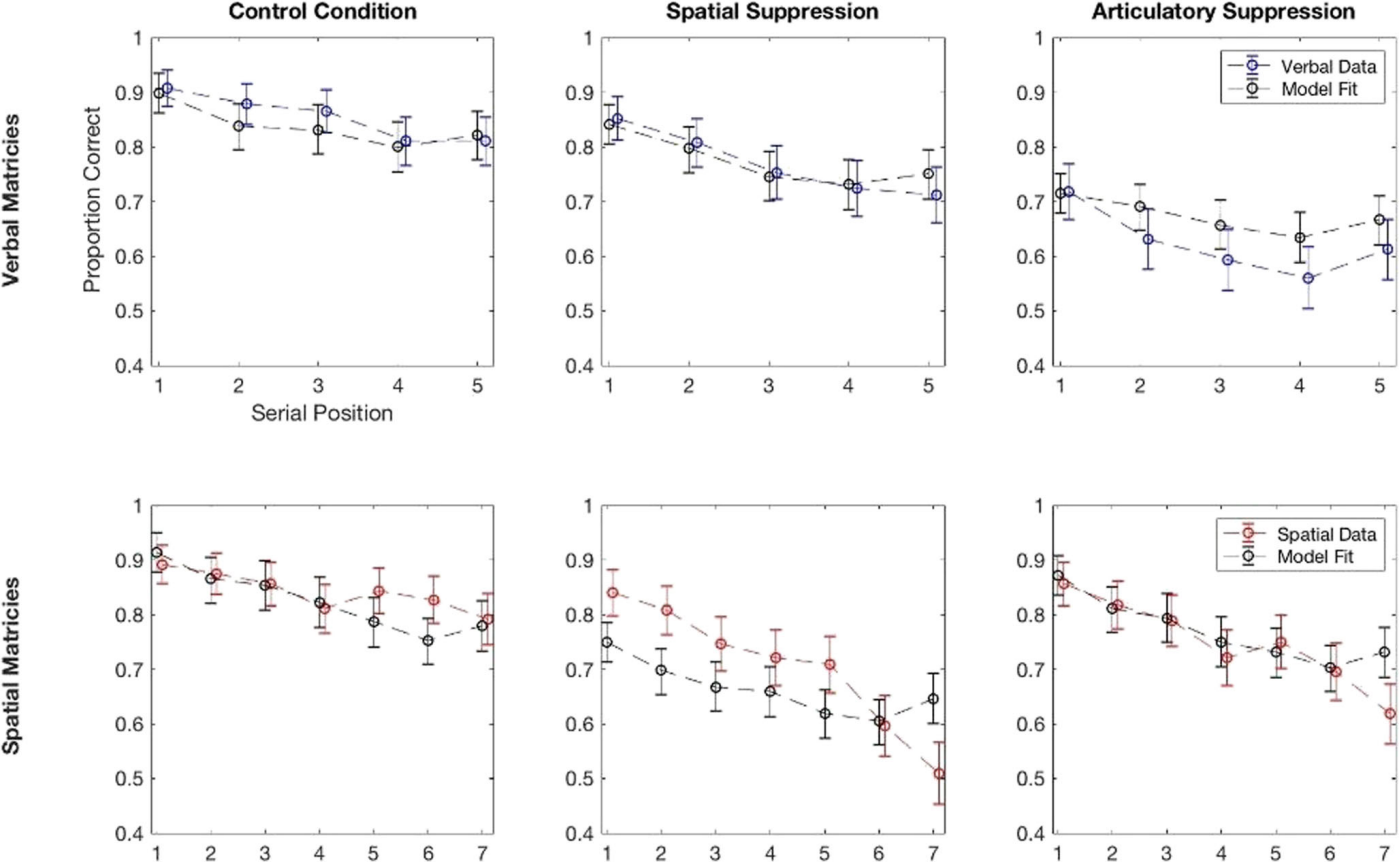
Data and model fits for each serial position and each condition. Error bars on data are ±2 SE, error bars on fits are estimates of 95% HDIs

First, for all conditions, we made the simplifying assumption that both tasks relied on traces holding the same number of modality dependent and independent features; this seemed justified as both tasks called upon the auditory modality and involved items that did not vary wildly in terms of complexity. Hence, for this first set of simulations, the only difference between the representations used for each primary task was in the number of items. As in the experiment, the “verbal trials” held five items, whereas the “spatial trials” had seven. We made no adjustments to the basic model’s operation to reflect the interval added to the primary tasks, although this was expected to have an impact on the recall of items in the recency positions (see Fig. 3).

We assumed that the two dual tasks tended to produce feature adoption at different rates *F*_*Tapping*_, *F*_*Suppression*_ and that feature adoption was enhanced by an additive factor *F*_*Dissociation*_ when dual and main tasks were similar. Values of *F*_*Dissociation*_ greater than zero tend to produce a dissociation, where, for example, articulatory suppression has a greater effect on verbal serial recall than on spatial recall. Together with the two attention parameters for the verbal and spatial conditions, *a*_*v*_, *a*_*s*_, this gave a total of five parameters. We also fit a null model where *F*_*Dissociation*_ was fixed to be zero. Further details of the fitting are given in the Appendix.

Figure 3 presents the results of the model fitting for the full model. The figure reproduces the serial position curve data from the experiment, together with some simulated data generated using the means of the posteriors for the model parameters. This simulated data also have error bars here since they are generated from a finite number of simulations (error bars are 95% HDIs estimated by bootstrapping).

As mentioned above, all six curves were modelled simultaneously with five free parameters. The final estimates for these parameters were as follows: distance parameter for the spatial task: 30.62 (95% HDI [22.20, 42.41]), distance parameter for the verbal stimuli: 25.51 (95% HDI [13.96, 31.74]), number of matching features induced by tapping : 9.14 (95% HDI [1.19, 18.00]), number of matching features induced by articulatory suppression: 10.59 (95% HDI [2.06, 18.58]), number of extra similar features when main and dual tasks match: 10.90 (95% HDI [0.55, 19.9]).

Overall, the fits appear to be reasonable and capture the main trends in the data. Some misfitting is evident, particularly in the spatial tapping case, but this data set is rather unusual in having no evidence for a recency effect; indeed, the final item is by far the least recalled. One notable issue is that the posteriors for the parameters are very broad, suggesting the model struggles to distinguish the effect of different values. Since the fits are fairly good, it is likely that a different choice of parameterization would produce tighter parameter estimates, but we did not explore this.

We can use a model comparison between the full and null models to assess the evidence for dissociations in this data. Using the same values of the ABC-PRC parameters as used in the main fits produced a Bayes Factor of 19.0 in favour of the full model. This is strong evidence for the superiority of the full model over the null one, and by extension, for the presence of a dissociation in this data.

In order to establish that the assumptions of the previous simulations could give a satisfactory approximation of another data set, a further set of model fits was obtained. The data were taken from the second experiment of Guérard and Tremblay (2008). In Experiment 2, participants completed a verbal order reconstruction task on half trials and a spatial order reconstruction task on the other half. In the verbal reconstruction task, seven words drawn from a closed pool of nine words were sequentially presented in the centre of a screen. At recall, all nine words reappeared simultaneously on the screen and participants were required to click on the seven studied items in the correct order. If they forgot an item, then they were to click on a question mark at the right of the screen. In the spatial order reconstruction task, on each trial seven dots were sequentially presented at various locations on the screen. The dots were randomly selected from a closed pool of nine dot locations. The recall procedure was the same as for the verbal task. One group of participants completed the verbal and spatial tasks alone and with articulatory suppression. In the articulatory suppression condition, participants were required to continuously repeat aloud the letters *A-B-C-D* during item presentation at the pace of two letters per second. The other group of participants completed the verbal and spatial tasks alone and with manual tapping. The tapping requirement and apparatus were the same as used in our experiment.

**Fig. 4:**
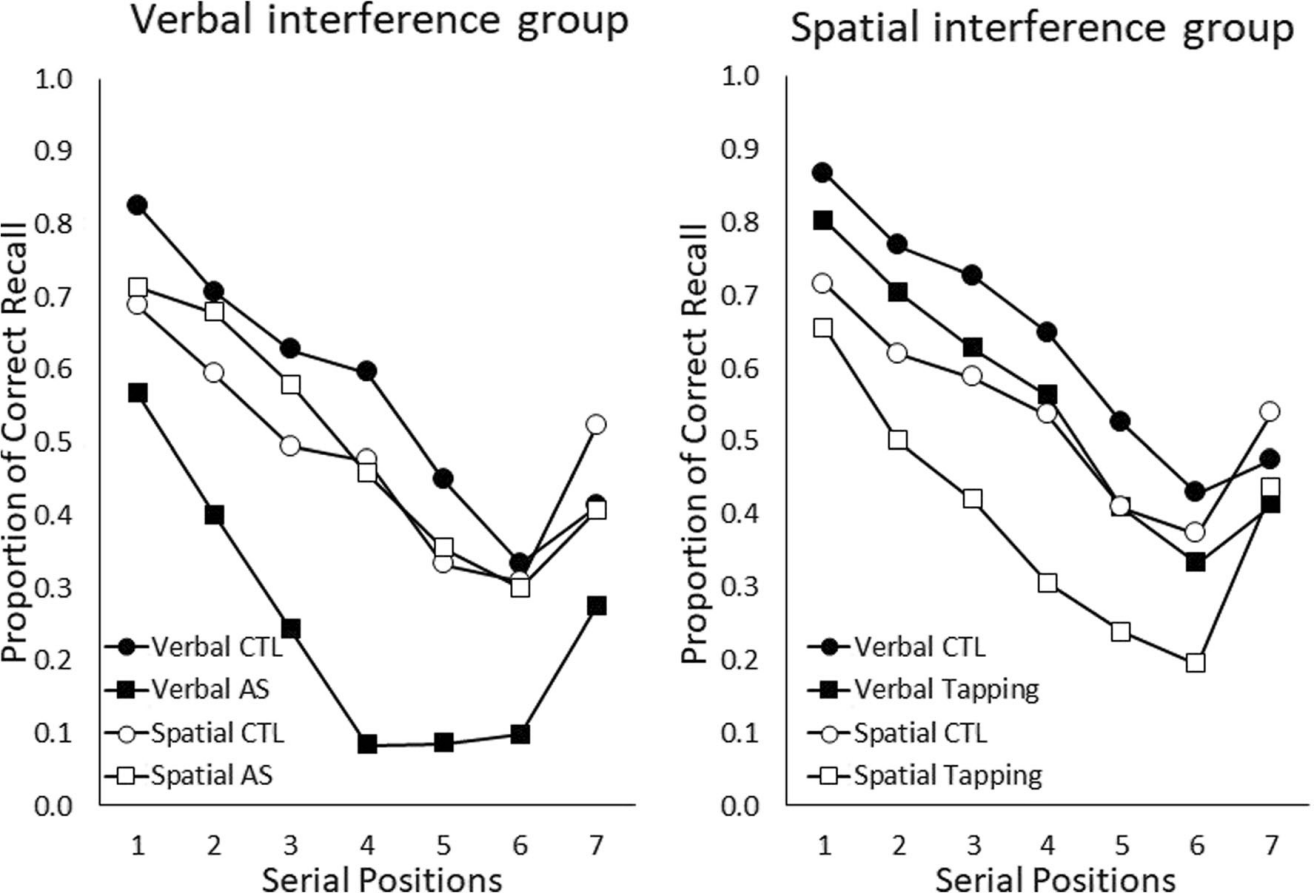
Proportion of correct recall as a function of serial position in the verbal and spatial serial order reconstruction tasks for the verbal interference group in the left panel and the spatial interference group in the right panel. CTL = control condition without suppression; AS = articulatory suppression; Tapping = manual tapping

Results of Guérard and Tremblay (2008) are presented in Fig. 4. There was a significant detrimental effect of articulatory suppression on the verbal order reconstruction task (*p* < .0001), but not on the spatial one (*p* = .51). However, a significant detrimental effect of manual tapping was reported with both the verbal (*p* = .001) and the spatial order reconstruction tasks (*p* < .0001).

### Fitting for Experiment 2 of Guérard and Tremblay (2008)

Model fitting was conducted in a similar way to Experiment 1 using ABC-PRC. However, since presentation was visual, we assumed two modality-dependent features and 20 modality-independent features, the typical setting for the FM (and the settings that have allowed the model to reproduce numerous benchmark effects). All conditions had seven items in this experiment. We made the decision to fit all eight conditions in the experiment at once, and to ignore the fact that some conditions are repeated. In a similar way to Experiment 1, we only allowed the distance scaling parameters and the number of similar features to vary.

We assumed that the two dual tasks tended to produce feature adoption at different rates *F*_*Tapping*_, *F*_*Suppression*_ and that feature adoption was enhanced by an additive factor *F*_*Dissociation*_ when dual and main tasks were similar. Values of *F*_*Dissociation*_ greater than zero tend to produce a dissociation, where, for example, articulatory suppression has a greater effect on verbal serial recall than on spatial recall. Together with the two attention parameters for the verbal and spatial conditions, *a*_*v*_, *a*_*s*_, this gave a total of five parameters. We also fit a null model where *F*_*Dissociation*_ was fixed to be zero.

**Fig. 5:**
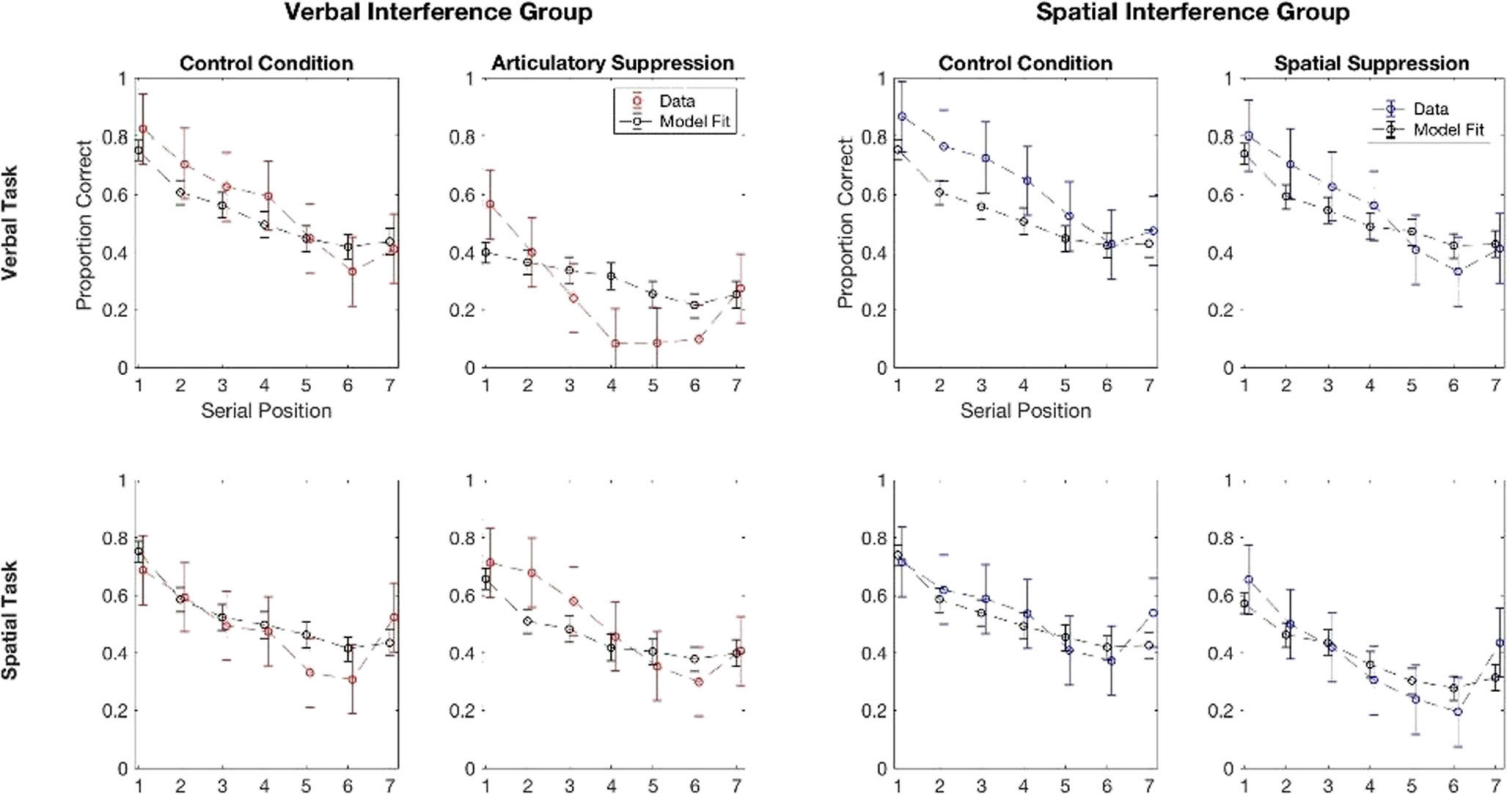
Data and model fits for each serial position and each condition. Error bars on data are ±2 *SE,* error bars on fits are estimates of 95% HDIs

Figures 5 presents the results of the model fitting for the full model. In a similar way to Fig. 3 we reproduce the serial position curve data from the experiment, together with some simulated data generated using the means of the posteriors for the model parameters. This simulated data also have error bars here since they are generated from a finite number of simulations (error bars are 95% HDIs estimated by bootstrapping).

The final estimates for the parameters were as follows: distance parameter for the spatial task: 29.60 (95% HDI [20.43, 42.36]), distance parameter for the verbal stimuli: 28.56 (95% HDI [18.81, 41.09]), number of matching features induced by tapping : 3.54 (95% HDI [0.20, 8.28]), number of matching features induced by articulatory suppression: 7.28 (95% HDI [1.80, 11.73]), number of extra similar features when main and dual tasks match: 7.57 (95% HDI [3.49, 12.43]).

Overall, the fits appear to be reasonable and capture the main trends in the data. The posteriors for the parameters are much tighter here, suggesting the model is finding it easier to distinguish the effects of different parameters. Some misfitting is evident, particularly for the verbal control conditions and the verbal suppression condition. Misfitting in the verbal control conditions is likely a consequence of the fact the model treats these conditions as identical, but there is some natural variability in the data, as there were two groups, each completing a verbal control condition. Misfitting in the verbal suppression condition is more interesting, and a consequence of the fact the FM struggles to fit serial position curves with a steep gradient.

We can use a model comparison between the full and null models in the same way as in Experiment 1 to assess the evidence for dissociations in this data. Using the same values of the ABC-PRC parameters as were used in the main fits produced a Bayes factor of >99 in favour of the full model. This is strong evidence for the superiority of the full model of the null one, and, by extension, for the presence of a dissociation in this data.

## General discussion

Double dissociations are often cited as evidence of separate systems (e.g. Norris, 2017; Schacter, 1987; Squire, 1994). Here, we asked if dissociations classically associated with the verbal and visuo-spatial components of the working-memory model (Baddeley, 2012; Baddeley & Hitch, 1974) can be accounted for within an alternative view that does not include dedicated verbal and visuo-spatial modules.

Our first experiment called upon the classic tasks developed by Brooks (1967). These verbal and visuo-spatial tasks were performed alone, in combination with a spatial suppression task or in combination with articulatory suppression. As expected, a single dissociation was observed. The spatial suppression task only affected the nonverbal version of the task while articulatory suppression influenced both primary tasks. The FM (Nairne, 1988, 1990; Neath, 1999; Neath & Nairne, 1995; Neath & Surprenant, 2007) was used to simulate these results. By assuming that the main difference between tasks (both primary and secondary) was in the encoded features, psychologically relevant changes to a few parameters generated a reasonable fit to the data. In a second series of simulations, the model was used to fit a more complex data pattern, involving a double dissociation, taken from Guérard and Tremblay (2008). To fit this data with the FM, we used the same set of parameters with one principled change to acknowledge the specific conditions tested.

The implications of the current work are straightforward; the type dissociations typically taken to support separate systems in the working memory literature can be accounted for by assuming differences in the features encoded for verbal and visuo-spatial materials, without assuming discrete structures. In other words, we called upon a computational model to show that variation in feature similarity, along with interference, are sufficient to produce single and double dissociations.

These results are generally in line with other observations that have insisted on the correspondence between memory of verbal and visuo-spatial materials. For instance, several authors have suggested that one of the central functions of short-term memory is related to serial order—and there is growing evidence that the mechanisms for this capacity apply across the verbal and visuo-spatial domains (Hurlstone, Hitch, & Baddeley, 2014; Majerus, 2009; C. C. Morey, 2018). In another example, Guérard, Neath, Surprenant, and Tremblay (2010) showed how distinctiveness effects in the spatial domain paralleled those reported with verbal material. These types of findings have led to the proposal of a unitary view of working memory—that is, one that does not include modality specific structures or modules (e.g. Macken, Tremblay, Alford, & Jones, 1999; Zimmer, 2009).

### Control processes

In the introduction, we mentioned the control processes highlighted in the original Atkinson and Shiffrin (1968) paper. The importance of the latter processes was significant in their framework. For instance, one section of their paper examined control processes involved in encoding; they offered the following: “It should be evident that there is a close relationship between the short- and long-term store. In general, information entering STS comes directly from LTS and only indirectly from the sensory register. For example, a visually presented word cannot be entered into STS as an auditory-verbal unit until a long-term search and match has identified the verbal representation of the visual image” (p. 115). We would like to argue that further work on control processes, in the sense of Atkinson and Shiffrin, could increase the power and flexibility of theoretical proposals, that is, their capacity to account for a variety of tasks as well as detailed patterns of results.

Consider the FM. Although the model can provide an explanatory framework for a sizable body of findings in the working-memory literature, it is a system focused on retrieval. Even simple tasks such as remembering a phone number or mental addition are likely to require dynamic processing not encompassed by the FM. As Atkinson and Shiffrin (1968) recognized, many tasks supported by short-term memory require flexible, task-related, control processes. To illustrate, let us consider the coding process potentially needed to perform the Brooks matrix tasks used here. In the visuo-spatial version, participants heard sentences that led them through an imagined path through a matrix (“In the starting square, put a 1, in the next square to the *right* put a 2, etc.”). Recall involved saying the words that described said path aloud (e.g. “right”, “down”, etc.). To perform this task, a coding control process must transform the auditory-verbal content to a visuo-spatial representation. Note that the items are presented in the *auditory modality,* but their content is *modality independent* (i.e. verbal instructions could be presented in the visual or auditory modality). The task facing the participants requires the development of a visuo-spatial representation—that is, a path within an imagined matrix. The FM assumes these operations take place and generate modality independent features; however, the model is silent with respect to how the necessary translation occurs. The same is true for the translation of visually presented words into categorized, meaning-laden, verbal-conceptual content. As in Atkinson and Shiffrin (1968), the suggestion here is that control processes accomplish this type of operation. To summarize, control processes could shed light on *how* features are generated and provide the flexibility necessary to generalize feature-based models to a wider variety of tasks—some of these control processes might equate to the modules originally proposed in the working-memory model (Baddeley, 2012; Baddeley & Hitch, 1974).

### Feature-based view relative to other proposals

An important question is what is achieved by shifting the focus from separate systems to a feature-based account. At a fundamental level, one can first ask if there are significant differences between these two approaches—after all, we know there are separate systems for the perception of auditory and visual stimuli, which must produce different features. Therefore, at some level, there are separate systems. Could the modular view and the feature-based view be more similar than they appear? These issues need to be carefully unpacked.

One point of import was alluded to earlier in the discussion. Although we know there are different systems for perceptual encoding, these modality differences are not the same as domain difference. In other words, differences between modalities (auditory/visual) do not equate to differences in verbal and visuo-spatial representations. Verbal and visuo-spatial information can be presented in more than one modality—that is, we can identify the *location* of a bird, a visuo-spatial item, by processing its song—and auditory cue. Similarly, we can identify a word’s rhyme, a verbal item, based on a printed presentation—a visual cue. So, separate modalities do not map one-to-one onto separate domains (verbal/visuo-spatial). By extension, one can argue that separate systems for different forms of perceptual information do not demand separate higher-order modules. Assuming retrieval is based on features that are not only perceptual, some integrated form of representation that includes the features generated by early modality-specific processing as well as by categorization and contact with prior knowledge, seems more likely to lead to efficient performance. The FM meets this requirement as it includes modality-specific as well as modality-independent features in the same representations.

What benefits might there be in adopting a feature-based view relative to a system proposing separated structures? One argument is that a feature-based working memory, without separate verbal and visuo-spatial modules, is more parsimonious—a point emphatically made by C. C. Morey (2018) in her recent review. We would suggest further benefits. First, principles that are very useful in other memory areas can be called upon much more easily in the working-memory domain when feature-based representations are viewed as central. For instance, consider the encoding-retrieval match principle. Encoding-retrieval match typically relates to multimodal, integrated features of to-be-remembered items, that is, features relating to item characteristics as well as the context in which the item appeared. Within a model that relies on feature-based representations, it is easy to see how encoding-retrieval match would apply. The same would be true of proposals such as encoding specificity. In effect, the Luce choice rule often included in feature-based views, can be seen as a quantitative instantiation of encoding specificity. Second, having a memory system where retrieval depends on the product of processing—the processes that generate the features—accords well with the need for multimodal integration of experience; sound, smells, feelings, thoughts, and visual properties can all be features of an encoded item. It seems less obvious how multimodal integration can be achieved within a modular system; some extra integrative module or structure would seem to be necessary.

## Conclusion

Since Atkinson and Shiffrin (1968) published their seminal paper, there has been a sizable amount of published work in the area of short-term/working memory. Multiple empirical phenomena have been uncovered and systematically studied, while computational and quantitative models have been proposed to explain these effects. Atkinson and Shiffrin illustrated how a systematic consideration of the experimental tasks bolstered by the precision of quantitative modelling can produce a compelling account. Their paper not only provided a very influential framework with respect to memory structure and processes, it illustrated how the combination of experimentation and modelling can be a very powerful approach.

Here, we tried to emulate this approach when considering dissociations between verbal and visuo-spatial working memory tasks. Our hypothesis regarding how these results could be understood was tested by quantitatively fitting the FM to two data sets. The demonstrations showed that both single and double dissociations can be explained within a system that does not include separate structures for verbal and visuo-spatial processing. The dissociations were accounted for by making the following assumptions: (1) verbal and visuo-spatial primary and secondary tasks generate different features; (2) suppression tasks produced traces that are integrated to the representations of to-be-recalled items in serial recall (the feature adoption hypothesis); (3) feature adoption or interference from secondary task is a function of differential similarity—when primary and secondary tasks share features, interference will be higher.

At a more general level, the work we reported is a further demonstration, within the working memory field, of the usefulness of feature-based approaches—something that Atkinson and Shiffrin (1968) identified. Many of the developments generated by their work involved more advanced feature-based models (i.e. search through associative memory [SAM], Raaijmakers & Shiffrin, 1980, 1981; retrieving effectively from memory [REM], Shiffrin & Steyvers, 1997; MINERVA 2, Hintzman, 1984; see Atkinson & Shiffrin, 2016, for further examples). The use made here of the FM is yet another example that shows that feature-based models can easily handle reasonably complex data patterns while relying on widely applicable principles.

### Author note

This research was supported in part by Grant No. 2015-04416 from the Natural Sciences and Engineering Research Council of Canada to Jean Saint-Aubin. While working on this article, Geneviève Gallant and Dominic Guitard were supported by NSERC graduate scholarships.

## Appendix: Model fitting details

Since the FM is too complex for an analytic expression for the likelihood to be derived, we used a version of approximate Bayesian computation (ABC) to carry out model fits (see Turner & Van Zandt, 2012; Marin et al. 2012, for a review). ABC methods allow for Bayesian model fitting even in cases when the likelihood cannot be computed, by using simulated data to obtain an approximate likelihood. Specifically, we used a procedure known as ABC partial rejection control (ABC-PRC; Sisson et al., 2007). ABC-PRC provides a good compromise between pure rejection sampling, which is simple but inefficient, and more sophisticated algorithms like ABC differential evolution (Turner & Sederberg, 2012), which can be more efficient but which are more complex.

ABC-PRC works by repeatedly sampling from a prior over the parameter space until it finds a set of parameters which generate a set of summary statistics sufficiently close to the data. When this happens, the algorithm stores these parameter values and moves on to the next particle in the generation. Once all particles in a generation have been associated with parameter sets, the algorithm gives each particle a weight depending on the prior, and then begins a new generation, sampling from the previous generation with probabilities given by the weights, and repeatedly perturbing around the previous parameter values until a set is found producing summary statistics even closer to the data. For full details, see Sisson et al. (2007; note also the errata, Sisson et al., 2009)

Under ABC-PRC, the posterior estimates for the parameters are just the fraction of particles in the final generation with that parameter value. Posterior predicted distributions of the summary statistics are also easily obtained.

The important parameters for ABC-PRC are the number of particles (set to 1,000 for all fits reported here), the number of generations (four here), the details of the prior, the proposal distributions, and the tolerances for each generation. Setting the number of generations and the tolerances requires some trial and error. Lower tolerances will tend to result in a better match between model and data, but at some point the computational cost becomes prohibitive. Details of the model and fitting parameters for each data set are given in Table 1.

**Table 1:** Fixed parameter values for all model fits reported in the main text

	Parameter values	Control conditions	
		Experiment 1	Guerard and Tremblay (2008)
Fixed model parameters	Forgetting probability	100	100
	Attention	1	1
	Recovery constant	2	2
	# of items	5 or 7	7
	***# independent features***	***20***	***20***
	Type ind. features	2	2
	***# dependent features***	***20***	***2***
	Type dep. features	2	2
Estimated Parameters	Distance scaling Parameters Mean [95% HDI]	*d*_*Spatial*_ = 30.62 [22.20, 42.41] *d*_*verbal*_ = 25.51 [13.96, 31.74]	*d*_*Spatial*_ = 29.60 [20.43, 42.36] *d*_*verbal*_ = 28.56 [18.81, 41.09]
	Number of similar features Mean [95% HDI]	*F*_*Tapping*_ = 9.14 [1.19, 18.00] *F*_*Suppression*_ = 10.59 [2.06, 18.58] *F*_*Dissociation*_ = 10.90 [0.55, 19.9]	*F*_*Tapping*_ = 3.54 [0.20, 8.28] *F*_*Suppression*_ = 7.28 [1.80, 11.73] *F*_*Dissociation*_ = 7.57 [3.49, 12.43]
Fitting Parameters	Number of particles	1000	1000
	Simulations per step	500	500
	Generations	4	4
	ABC parameters	*ϵ* = [.15 .10 .08 .06] Proposal *λ* = [5 12 14 20] Proposal *SD* = [ 5 3 2 2]	*ϵ* = [.18 .15 .12 .10] Proposal *λ* = [5 12 14 20] Proposal *SD* = [5 3 2 2]

*Note.* The majority of these parameter values are identical to those used in the simulations in Neath and Nairne (1995)

To assess the presence of a dissociation we compared the full model with a reduced, or null, model where the number of extra similar features induced when the modality of the dual task matched the primary was set to be zero. To do this, we make use of a standard trick (Marin et al., 2012), which is to imagine the two models as nested in some larger model which includes a parameter *m* that selects one model or the other. We can run a simple rejection sampling algorithm on this joint model to estimate the value of *m*. Assuming a uniform prior, the ratio of the posterior probabilities, *p*(*m* = *full*)/*p*(*m* = *null*) is an estimate of the Bayes factor *BF*. In both experiments, we ran this rejection sampling step at the lowest value of epsilon used in the main fitting; however, since this is computationally inefficient compared with ABC-PRC, we used only 100 particles. Quoted Bayes factors are therefore to be regarded as approximations. The data and model code used in our analysis are available on the Open Science Framework project page (https://osf.io/6sae4/)
